# Development of an In-Patient Satisfaction Questionnaire for the Chinese Population

**DOI:** 10.1371/journal.pone.0144785

**Published:** 2015-12-11

**Authors:** Jie Wei, Xin-liang Wang, Hao-bin Yang, Tu-bao Yang

**Affiliations:** Department of Epidemiology and Health Statistics, School of Public Health, Central South University, Changsha, Hunan Province, China; University of Chieti, ITALY

## Abstract

**Background:**

Patients’ satisfaction has been considered as a crucial measurement of health care quality. Our objective was to develop a reliable and practical questionnaire for the assessment of in-patients’ satisfaction in Chinese people, and report the current situation of in-patients’ satisfaction in the central south area of China through a large-scale cross-sectional study.

**Design:**

In order to generate the questionnaire, we reviewed previous studies, interviewed related people, held discussions, refined questionnaire items after the pilot study, and finally conducted a large cross-sectional survey to test the questionnaire.

**Setting:**

This study was conducted in three A-level hospitals in the Hunan province, China.

**Results:**

There were 6640 patients in this large-scale survey (another 695 patients in the pilot study). A factor analysis on the data from the pilot study generated four dimensions, namely, doctors’ care quality, nurses’ care quality, quality of the environment and facilities, and comprehensive quality. The Cronbach’s alpha coefficients for each dimension were above 0.7 and the inter-subscale correlation was between 0.72 and 0.83. The overall in-patient satisfaction rate was 89.6%.

**Conclusion:**

The in-patient satisfaction questionnaire was proved to have optimal internal consistency, reliability, and validity.

## Introduction

Patients’ satisfaction is considered to be a measure of health care, and hospitals worldwide use it to improve the quality of health care. [1,2] In the past, we used to evaluate the quality of medical services by evaluating the objective outcomes of patients’ physical condition. Recently however, researchers have begun to pay close attention to patients’ satisfaction as a yardstick for assessing the effectiveness and quality of medical care. [3] Although the quality of medical services can be evaluated by multiple perspectives, such as doctors, patients or insurer, patients should still be considered as the most important estimator of the quality of care. [4] Patients' opinions and satisfaction status may affect their future behaviors related to the treatment outcomes. [4,5] Analysis of the patients’ subjective feedback can fully understand the areas that need to be improved, which can upgrade the quality of medical care. [6–8]

As a result of the increasing value of patients’ satisfaction, various kinds of measurement tools are being developed and tested. Suggestion boxes, formal complaints, qualitative methods, audits, and satisfaction questionnaires are being used to assess the level of patients’ satisfaction, the satisfaction questionnaire being the most effective and widely used method. [9]

In the last decade, a large number of questionnaires targeting all kinds of patients and different areas of medical care have been developed, especially in well-developed countries. [10–14] However, some of them are criticized for their poor validity and reliability. [6,15] Furthermore, the definition of patients’ satisfaction has sometimes been misunderstood, leading to exceedingly high ratings of patients’ satisfaction.[16,17] More importantly, there has been no large sample research to fully test an in-patients’ satisfaction questionnaire on the Chinese population. Unlike most of the developed countries, we have a special medical environment (scarce or unbalanced medical resources) and large population. Therefore, questionnaires developed in these countries may not be suitable for use in China as well as in other developing countries.

In an earlier study, we developed the Chinese outpatient satisfaction questionnaire (Ch-OPSQ).[18] The objective of the present study was to develop an in-patients’ satisfaction questionnaire for the Chinese population, and to test the reliability, validity, and acceptability of the self-administrated questionnaire on a large cross-sectional sample.

## Method

### Study Population

First, a pilot study involving 695 patients was performed in one teaching hospital using a 41 items questionnaire. Then, on the basis of the results of the pilot survey, a following cross-sectional study on three A-level hospitals in the Hunan province of China, including 6640 patients and using a final version of 28 items questionnaire, was performed.

### The draft questionnaire

Two members from our team reviewed previously published studies. The Medline and Embase databases were searched using the following key words: “patients,” “hospital,” “satisfaction,” and “questionnaire.” We screened all the relevant studies and extracted useful information. The information were mainly the evaluated dimensions and items from existed inpatients satisfaction questionnaire developed by other countries. Then the research group discussed which items to choose for developing our questionnaire. Evaluating items related to the treatment process, medical provider (doctors and nurses), and hospitalization environment were considered to be included in the questionnaire. Evaluating items were not suitable for the Chinese medical condition, such as reservation process, cultural difference and medical insurance system, were excluded from the questionnaire.

### A modified version of the questionnaire and pilot survey

The research group interviewed five patients, five administrators of different hospitals and five officers from the health department of the Government about the draft questionnaire. According to patients’ admission number and job number, simple random sampling method were applied to select these interviewees. Consultations with these participants were primarily held face-to-face or via email. All the interviewees were asked to rate the importance of each item, provide their opinions and suggestions about the items in the item pool, comment on the relevance of the issues covered, and comprehensibility of the questionnaire and response options. The Research group reviewed the suggestions from the interviews, refined the wording and content of the questions in the draft questionnaire, and build consensus on the items and response options according to their feedback. Then a modified version of the questionnaire was created for a pilot study.

The pilot survey was conducted in one teaching hospital using the modified version of the questionnaire, which contained 41 items, including patients’ basic information and the rating of their feelings about each statement on a 5-point Likert scale: very satisfied (= 5), relatively satisfied (= 4), fairly satisfied (= 3), relatively dissatisfied (= 2) and very dissatisfied (= 1) (Table A in S1 File). We handed out the questionnaires to patients in their sickrooms and collected them after they had completed them. After analyzing the collected data, a discussion was held and the research group further discussed the selection of the items. Some items were excluded because of the high non-response rate (such as “security's service”; “food from hospital cafeteria”; “the opportunity of asking for the medication condition of yourself”; “the right to know your medication decision”; “introduction of the ward environment and points for attention”). And some items were excluded based on the similarity with other items and the responses showed poor variability, they were amended and merged with others (such as “polite language usage by doctors”; “the initiative of explanation of the medication by nurses”; “explanation of the side effect of the medication”; “how well nurses cared about your pain and uncomfortable feelings”; “how well nurses responded to your complains”). We also excluded items based on the principal component exploratory factor analysis. Items with poor factor loadings were considered to be excluded (such as “daily medical cost”; “Disease improvement“). Thus, the final version of the questionnaire including 28 items was generated (Table B in S1 File).

### The cross-sectional study on a large sample

We sent the final version of the questionnaire to three A-level hospitals in the Hunan province of China for further evaluation. Additionally, the current situation of in-patients’ satisfaction in the central south area of China was evaluated. Conscious patients who had stayed in the hospital for over three days were randomly selected for this satisfaction survey. The research group trained twenty investigators and sent them to the chosen hospitals. The investigators were all from a third party institution that was not related to these hospitals. All patients independently completed the questionnaires in their sickroom. The research was approved by the Ethics Committee of Central South University. Written informed consent was obtained from all the subjects in this study. A total of 6640 patients were included in the cross-sectional study, only 4618 patients’ basic characteristics were recorded. The data of 1822 patients were missing because these were pediatric patients, whose satisfaction level was rated by their parents.

### Statistical analysis

The number and frequency for categorical variables, and the mean and standard deviation for the continuous variables, were calculated as descriptive statistics. Construct validity refers to the extent to which the new questionnaire conforms to existing ideas or hypotheses concerning the concepts that are being measured. [19,20] A principal component exploratory factor analysis by varimax rotation was used to establish the structure and test the construct validity of the questionnaire. Factors with an eigenvalue greater than one or a cumulative contribution rate of above 70% were extracted. Items were included in the dimensions only if they revealed loadings greater than 0.4 after rotation. Items with poor factor loadings were considered for removal from the final questionnaire. Further, if items showed multiple loadings above 0.4, they were included in the factor with which they had a better conceptual relationship.[21] The hypothesis was that it is possible to obtain meaningful, independent, and efficient dimensions to evaluate patient satisfaction.

Reliability indicates a consistency of the performance on the questionnaire. Good reliability produces similar results under consistent conditions.[19]The internal consistency and reliability of each dimension was examined by the Cronbach’s alpha and inter-subscale correlations. The Cronbach’s alpha assesses the overall correlation between items within a scale. An alpha value of 0.7 or higher is recommended as an indicator of sufficient reliability of a scale. Additionally, in order to prove the independency of each dimension, the inter-subscale correlation should be lower than the corresponding Cronbach’s alpha.[22]

The feasibility and acceptability of the tool refers to the ease of use of the questionnaire.[23] They were examined by the percentage of missing item responses, interviewer-reported acceptability, and the time and ease of administration. Finally, the score and satisfaction level were reported. The satisfaction rate was calculated in accordance with the following formulae from previous studies [18,24]:

$$\text{Satisfied rate}=\frac{\text{mean score}}{5}*100\%\;(\text{for a single item})$$

$$\text{Satisfied rate}=\frac{\text{mean score}}{5*\text{number of items}}*100\%\;(\text{for the dimensions and overall satisfied rate})$$

A multiple logistic regression analysis was conducted to identify whether the potential determinants such as patients’ age, sex, occupation, education background, and medical insurance type were significantly associated with the overall satisfaction as the dependent variable.

Patients’ responses were entered into the Epidata 3.1, and the data analysis was subsequently performed using the SPSS 17.0. A *P* value of less than 0.05 was considered to be statistically significant.

## Results

The pilot study included 695 patients. The data from the pilot study were gathered for assessing the quality of the questionnaire. The 28 items that were related to the quality of medical care were included in the factor analysis. The results indicated that four factors with eigenvalues greater than one explained 73.7% of the variance. These were Doctors’ care quality (9 items), Nurses’ care quality (12 items), Quality of the environmentand facilities’ (5 items), and Overall medical quality (2 items). The final items have been listed in Table 1.

**Table 1:** Factor analysis loadings for the pilot study (n = 695).

Item number	Content	Factor1*loading	Factor2*loading	Factor3*loading	Factor4*loading
1	The duration of your wait-time for a doctor after admission	0.64			
2	The extent to which doctors respected your privacy during your stay	0.57			
3	Reviewing your medical history	0.70			
4	How well doctors met your requirements	0.67			
5	Diagnosis and treatment provided to you	0.75			
6	Courtesy of the doctors	0.79			
7	Explanation of the purpose of discharge	0.71			
8	How well doctors responded to your health care problems	0.75			
9	Doctors’ ward-rounds	0.73			
10	The attitude of the admission staff		0.68		
11	Explanation of the hospital routine and procedures		0.73		
12	Duration of wait-time for a bed (after you arrived at the hospital)		0.67		
13	Explanation of the medication by nurses		0.73		
14	Courtesy of the nurses		0.68		
15	Nurses’ ward-rounds		0.76		
16	The extent to which nurses respected your privacy during your stay		0.68		
17	Making your beds		0.73		
18	The quality of the care provided by nurses		0.69		
19	How well nurses responded to your health care problems		0.58		
20	Nurses’ medical techniques		0.64		
21	Duration of wait-time for a nurse after using the call system		0.73		
22	The restfulness of the hospital (amount of peace and quiet)			0.81	
23	The cleanliness of the toilets and showers			0.80	
24	Your comfort during your stay			0.82	
25	The privacy in the room where you spent the most time			0.62	
26	The cleanliness of patients’ clothes			0.62	
27	Global assessment of the medical quality				0.73
28	Global assessment of the service quality				0.72

Only loadings greater than 0.4 have been listed.

*: Factor 1, doctors’ care quality; Factor 2, nurses’ care quality; Factor 3: Quality of the environmentand facilities, and Factor4, comprehensive quality.

The large-scale cross-sectional study on a sample of 6640 in-patients was conducted from July 2012 to July 2013. The demographic characteristics of 4618 patients were recorded. Among the 4618 patients, 2256 were men (48.9%), and the mean age of the sample was 45.1 years. Further, 52.5% of them lived in rural areas, 33.7% of them were famers, and 30.3% of them had only studied up to middle school. Nearly half of them (48%) were covered under the rural cooperating medical insurance (Table 2).

**Table 2:** Patients’ demographic characteristics (n = 4618).

	Mean(SD)	N	%
Age	45.1(19.3)		
Sex			
Male		2256	48.9
Female		2264	49.0
Missing		98	2.1
Residence			
Rural area		2420	52.4
Urban area		2158	46.7
Missing		40	0.9
Occupation			
Worker		594	12.9
Farmer		1553	33.6
Civil servant		437	9.5
Medical worker		106	2.3
Merchant		471	10.2
Other		1419	30.7
Missing		38	0.8
Education background			
Primary school or lower		828	17.9
Middle school		1398	30.3
High school		1230	26.6
College or higher		1115	24.1
Missing		47	1.1
Medical insurance			
Rural cooperating medical insurance		2213	47.9
Urban workers’ medical insurance		1397	30.3
Urban residents’ medical insurance		566	12.3
No medical insurance		219	4.7
Other medical insurance		175	3.8
Missing		48	1.0

We further confirmed the reliability of the final version of the satisfaction questionnaire. In each dimension, the Cronbach’s alpha was above 0.8 for all the items and the inter-subscale correlation was between 0.722 and 0.841. (Table 3)

**Table 3:** Internal consistency scores for the final version of the questionnaire.

	Doctors’ care quality	Nurses’ care quality	Quality of the environmentand facilities’	Comprehensive quality
Doctors’ care quality	**0.955**			
Nurses’ care quality	0.841	**0.959**		
Quality of the environment and facilities’	0.722	0.733	**0.906**	
Comprehensive quality	0.776	0.740	0.750	**0.816**

The numbers in bold represent the Cronbach’s alpha coefficients.

According to the results (Table 4), patients were the most satisfied with bed making (satisfaction rate was 92%) and the least satisfied with the restfulness of the hospital and cleanliness of the toilets and showers (satisfaction rate was 82%). Comparing the four dimensions, the highest satisfaction rate was observed for the doctors’ care quality (87.6%), while the same was the lowest for the quality of the environment and facilities’ (83.2%). The overall satisfaction, evaluated by all 28 items, was 89.6%. The results of the multiple logistic regression suggested that age, occupation, educational background, and medical insurance were the determinants of the patients’ overall satisfaction rate (Table 5). Specifically, the satisfaction rate was higher in patients who were older (OR = 1.12), who were covered under the urban workers’ medical insurance (as compared to those covered under the rural cooperating medical insurance) (OR = 1.21), those with higher education (OR = 1.2), and those who were farmers (as compared with those who were workers) (OR = 0.76).

**Table 4:** Satisfaction characteristics.

Item number	Content	Mean	Standard deviation	Satisfaction rate (%)
1	The duration of your wait-time for a doctor after admission	4.3	0.7	86
2	The extent to which doctors respected your privacy during your stay	4.5	0.7	90
3	Reviewing your medical history	4.4	0.8	88
4	How well doctors met your requirements	4.3	0.8	86
5	Diagnosis and treatment provided to you	4.5	0.8	90
6	Courtesy of the doctors	4.5	0.8	90
7	Explanation of the purpose of discharge	4.4	0.8	88
8	How well doctors responded to your health care problems	4.3	0.9	86
9	Doctors’ ward-rounds	4.4	0.8	88
10	The attitude of the admission staff	4.3	0.8	86
11	Explanation of the hospital routine and procedures	4.4	0.8	88
12	Duration of wait-time for a bed (after you arrived at the hospital)	4.3	0.8	86
13	Explanation of the medication	4.3	0.9	86
14	Courtesy of the nurses	4.4	0.8	88
15	Nurses’ ward-rounds	4.4	0.8	88
16	The extent to which nurses respected your privacy during your stay	4.5	0.7	90
17	Making your beds	4.6	0.7	92
18	The quality of the care provided by nurses	4.3	0.9	86
19	How well nurses responded to your health care problems	4.3	0.8	86
20	Nurses’ medical techniques	4.4	0.8	88
21	Duration of wait-timefor a nurse after using the call system	4.3	0.9	86
22	The restfulness of the hospital (amount of peace and quiet)	4.1	0.9	82
23	The cleanliness of the toilets and showers	4.1	1.0	82
24	Your comfort during your stay	4.2	0.9	84
25	The privacy in the room where you spent the most time	4.2	0.9	84
26	The cleanliness of patients’ clothes	4.2	0.9	84
27	Global assessment of the medical quality	4.2	1.0	84
28	Global assessment of the service quality	4.4	0.7	88
Doctors’ care quality (9 items)		39.4	6.2	87.6
Nurses’ care quality (12 items)		52.3	8.1	87.2
Quality of the environment and facilities’ (5 items)		20.8	3.9	83.2
Global quality (2 items)		8.5	1.53	85
Total (28 items)		125.5	18.8	89.6

**Table 5:** Results of a multiple logistic regression (the independent variables were selected by the backward method).

Independent variables	β	Standard error	P	OR
Age^#^	0.11	0.024	0.000	1.120
Occupation (as compared with workers)				
Farmer	-0.281	0.142	0.048	0.755
Other	-0.186	0.083	0.025	0.830
Education background (as compared withthose educated up to middle school)				
High school	0.179	0.097	0.066	1.196
Medical insurance (as compared with those covered under therural cooperative medical insurance)				
Urban workers’ medical insurance	0.192	0.094	0.041	1.212

#: Age was converted into categorical data as younger than 20 years, 21 to 30 years,31 to 40 years, 41 to 50 years, 51 to 60 years, and older than 61 years.

## Discussion

Our research group developed a Chinese self-administered questionnaire on in-patients’ satisfaction. Patients rated their satisfaction level according to their experience regarding several important aspects of medical treatment. The results from the pilot survey and large-scale survey indicated that the in-patients’ satisfaction questionnaire had optimal quality.

The questionnaire was subject to a series of testing processes to assess its reliability and validity. Four main dimensions of the questionnaire were similar to the tools used in previous studies.[16,25–29] Moreover, the interpretation of the dimensions was verified in another study.[30] The Cronbach’s alpha coefficients of the four dimensions were all above the recommended minimum of 0.7, [31] the inter-subscale correlations were lower than the internal consistency for each scale. These outcomes indicated that the reliability of the too, as indicated by the internal consistency, was excellent and these findings were consistent with those of other studies [19,27,28].[31–33] The questionnaire also had good acceptability. The core items related to medical care quality had a high response rate (higher than 99.7%), and the patients could complete the questionnaire within ten to fifty minutes, which showed perfect acceptability and feasibility.

According to the large-scale survey, the overall satisfaction rate was 89.6%. Our study showed that the satisfaction related to doctors’ care quality was the highest, while that on the quality of the environment and facilities’ was the lowest. These results concurred with those presented in other studies.[32,34,35] Results of a logistic regression identified that age, occupation, education background, and type of medical insurance of the patients could be determinants of their overall satisfaction. Several studies [26,32,34] concluded that younger patients had a lower satisfaction rate. Hali, J. et al [26] conducted a meta-analysis on the determinants of patients’ satisfaction, which revealed that education and social status could predict patients’ satisfaction. Kats M. et al [36] suggested that patients’ satisfaction was associated with their medical insurance type among HIV-Infected men. These were consistent with our results too. Compared with most standard instruments developed in North America and the UK, where the surveys were conducted after hospitalization, we collected our data while the patients were in the hospital. Recently, the Hong Kong (HK) government conducted a thematic household survey using the Piker patient experience questionnaire-15 (PPE-15) to measure in-patients’ satisfaction.[37] The survey revealed an overall satisfaction rate of 77.9%,[38] which was lower than our findings. The HK Hospital Authority (HA), an independent public sector organization, developed a patients’ experience tool named HK Inpatient Experience Questionnaire (HKIEQ) in 2009.[39] However, the medical care system of HK is very different from that of mainland China. In addition, our survey employed a larger sample as compared to that in previous studies. We also developed a CH-OPSQ in a previous study.[18] Both CH-OPSQ and inpatients satisfaction questionnaire went through a strict development process and were tested to be with good reliability and validity. The mainly differences between the OPSQ and the inpatients satisfaction questionnaire were the structure of the questionnaire and the satisfaction outcomes. There were 6 dimensions (waiting time, service attitude, medical care quality, special service quality, environment quality, global assessment) in CH-OPSQ due to the complicated organization of outpatient services comparing to 4 dimensions in the inpatients satisfaction questionnaire. In addition, for satisfaction outcomes, the waiting time appears to be a major issue for outpatients. But in the present study, the quality of the environment and facilities was with the lowest satisfaction for inpatients.

The first limitation of our study is that we did not evaluate the test-retest reliability of the tool. However, this was not feasible as most of the participants lived in rural areas and the communication methods were limited. Future research should focus on testing the test-retest reliability of our instrument using accept techniques. Another possible limitation is that we gathered background information only from 4618 patients. The data of 1822 patients were missing because these were pediatric patients, whose satisfaction level was rated by their parents. It was almost impossible to collect complete information of the demographic characteristics of these patients. For example, they did not have a job and some of them were too young to be educated. Therefore, we chose not to record their basic information.

In conclusion, the in-patients’ satisfaction questionnaire developed in this study had optimal validity, reliability, and acceptability. Additionally, the in-patient satisfaction in the central south area of China was relatively high in terms of the medical processes and relatively low in terms of the hospital environment and comfort. Finally, age, occupation, educational background, and type of medical insurance of the patients were the determinants of patients’ overall satisfaction rate.

## Supporting Information

S1 File Inpatients satisfaction questionnaire for pilot study (41 items) (Table A). Final version of the inpatients satisfaction questionnaire (28 items) (Table B).
(DOCX)
