# Supplementary material for: Development of an In-Patient Satisfaction Questionnaire for the Chinese Population
Source: PLoS One. 2015 Dec 11;10(12):e0144785. doi: 10.1371/journal.pone.0144785 (PMC4684244; doi:10.1371/journal.pone.0144785)
Supplement: S1 File — Final version of the inpatients satisfaction questionnaire (28 items) (Table B). (DOCX) [file pone.0144785.s001.docx]

Table A. Inpatients satisfaction questionnaire for pilot study (41 items)

| Item number | Content | Very satisfied | Relatively satisfied | Fairly satisfied | Relatively dissatisfied | Very dissatisfied |
| --- | --- | --- | --- | --- | --- | --- |
| 1 | The duration of your wait-time for a doctor after admission | 5 | 4 | 3 | 2 | 1 |
| 2 | Polite language usage by doctors | 5 | 4 | 3 | 2 | 1 |
| 3 | The extent to which doctors respected your privacy during your stay | 5 | 4 | 3 | 2 | 1 |
| 4 | Reviewing your medical history | 5 | 4 | 3 | 2 | 1 |
| 5 | How well doctors met your requirements | 5 | 4 | 3 | 2 | 1 |
| 6 | Diagnosis and treatment provided to you | 5 | 4 | 3 | 2 | 1 |
| 7 | Courtesy of the doctors | 5 | 4 | 3 | 2 | 1 |
| 8 | Explanation of the purpose of discharge | 5 | 4 | 3 | 2 | 1 |
| 9 | How well doctors responded to your health care problems | 5 | 4 | 3 | 2 | 1 |
| 10 | Doctors’ ward-rounds | 5 | 4 | 3 | 2 | 1 |
| 11 | The attitude of the admission staff | 5 | 4 | 3 | 2 | 1 |
| 12 | Explanation of the hospital routine and procedures | 5 | 4 | 3 | 2 | 1 |
| 13 | Duration of wait-time for a bed (after you arrived at the hospital) | 5 | 4 | 3 | 2 | 1 |
| 14 | Introduction of the ward environment and points for attention | 5 | 4 | 3 | 2 | 1 |
| 15 | Explanation of the medication by nurses | 5 | 4 | 3 | 2 | 1 |
| 16 | Explanation of the side effect of the medication | 5 | 4 | 3 | 2 | 1 |
| 17 | Courtesy of the nurses | 5 | 4 | 3 | 2 | 1 |
| 18 | Polite language usage by nurses |  |  |  |  |  |
| 19 | Nurses’ ward-rounds | 5 | 4 | 3 | 2 | 1 |
| 20 | The extent to which nurses respected your privacy during your stay | 5 | 4 | 3 | 2 | 1 |
| 21 | Making your beds | 5 | 4 | 3 | 2 | 1 |
| 22 | The quality of the care provided by nurses | 5 | 4 | 3 | 2 | 1 |
| 23 | How well nurses responded to your health care problems | 5 | 4 | 3 | 2 | 1 |
| 24 | How well nurses cared about your pain and uncomfortable feelings | 5 | 4 | 3 | 2 | 1 |
| 25 | How well nurses responded to your complains | 5 | 4 | 3 | 2 | 1 |
| 26 | Nurses’ medical techniques | 5 | 4 | 3 | 2 | 1 |
| 27 | Duration of wait-time for a nurse after using the call system | 5 | 4 | 3 | 2 | 1 |
| 28 | The opportunity of asking for the medication condition of yourself | 5 | 4 | 3 | 2 | 1 |
| 29 | The right to know your medication decision | 5 | 4 | 3 | 2 | 1 |
| 30 | The restfulness of the hospital (amount of peace and quiet) | 5 | 4 | 3 | 2 | 1 |
| 31 | The cleanliness of the toilets and showers | 5 | 4 | 3 | 2 | 1 |
| 32 | Your comfort during your stay | 5 | 4 | 3 | 2 | 1 |
| 33 | The privacy in the room where you spent the most time | 5 | 4 | 3 | 2 | 1 |
| 34 | The cleanliness of patients’ clothes | 5 | 4 | 3 | 2 | 1 |
| 35 | Guiding facilities | 5 | 4 | 3 | 2 | 1 |
| 36 | Security's service | 5 | 4 | 3 | 2 | 1 |
| 37 | Food from hospital cafeteria | 5 | 4 | 3 | 2 | 1 |
| 38 | Daily medical costs | 5 | 4 | 3 | 2 | 1 |
| 39 | Disease improvement | 5 | 4 | 3 | 2 | 1 |
| 40 | Global assessment of the medical quality | 5 | 4 | 3 | 2 | 1 |
| 41 | Global assessment of the service quality | 5 | 4 | 3 | 2 | 1 |

Table B. Final version of the inpatients satisfaction questionnaire (28 items)

| Item number | Content | Very satisfied | Relatively satisfied | Fairly satisfied | Relatively dissatisfied | Very dissatisfied |
| --- | --- | --- | --- | --- | --- | --- |
| 1 | The duration of your wait-time for a doctor after admission | 5 | 4 | 3 | 2 | 1 |
| 2 | The extent to which doctors respected your privacy during your stay | 5 | 4 | 3 | 2 | 1 |
| 3 | Reviewing your medical history | 5 | 4 | 3 | 2 | 1 |
| 4 | How well doctors met your requirements | 5 | 4 | 3 | 2 | 1 |
| 5 | Diagnosis and treatment provided to you | 5 | 4 | 3 | 2 | 1 |
| 6 | Courtesy of the doctors | 5 | 4 | 3 | 2 | 1 |
| 7 | Explanation of the purpose of discharge | 5 | 4 | 3 | 2 | 1 |
| 8 | How well doctors responded to your health care problems | 5 | 4 | 3 | 2 | 1 |
| 9 | Doctors’ ward-rounds | 5 | 4 | 3 | 2 | 1 |
| 10 | The attitude of the admission staff | 5 | 4 | 3 | 2 | 1 |
| 11 | Explanation of the hospital routine and procedures | 5 | 4 | 3 | 2 | 1 |
| 12 | Duration of wait-time for a bed (after you arrived at the hospital) | 5 | 4 | 3 | 2 | 1 |
| 13 | Explanation of the medication by nurses | 5 | 4 | 3 | 2 | 1 |
| 14 | Courtesy of the nurses | 5 | 4 | 3 | 2 | 1 |
| 15 | Nurses’ ward-rounds | 5 | 4 | 3 | 2 | 1 |
| 16 | The extent to which nurses respected your privacy during your stay | 5 | 4 | 3 | 2 | 1 |
| 17 | Making your beds | 5 | 4 | 3 | 2 | 1 |
| 18 | The quality of the care provided by nurses | 5 | 4 | 3 | 2 | 1 |
| 19 | How well nurses responded to your health care problems | 5 | 4 | 3 | 2 | 1 |
| 20 | Nurses’ medical techniques | 5 | 4 | 3 | 2 | 1 |
| 21 | Duration of wait-time for a nurse after using the call system | 5 | 4 | 3 | 2 | 1 |
| 22 | The restfulness of the hospital (amount of peace and quiet) | 5 | 4 | 3 | 2 | 1 |
| 23 | The cleanliness of the toilets and showers | 5 | 4 | 3 | 2 | 1 |
| 24 | Your comfort during your stay | 5 | 4 | 3 | 2 | 1 |
| 25 | The privacy in the room where you spent the most time | 5 | 4 | 3 | 2 | 1 |
| 26 | The cleanliness of patients’ clothes | 5 | 4 | 3 | 2 | 1 |
| 27 | Global assessment of the medical quality | 5 | 4 | 3 | 2 | 1 |
| 28 | Global assessment of the service quality | 5 | 4 | 3 | 2 | 1 |
